# Ochratoxin A—The Current Knowledge Concerning Hepatotoxicity, Mode of Action and Possible Prevention

**DOI:** 10.3390/molecules28186617

**Published:** 2023-09-14

**Authors:** Magdalena Więckowska, Rafał Szelenberger, Marcin Niemcewicz, Piotr Harmata, Tomasz Poplawski, Michał Bijak

**Affiliations:** 1 Biohazard Prevention Centre, Faculty of Biology and Environmental Protection, University of Lodz, Pomorska 141/143, 90-236 Lodz, Poland; magdalena.wieckowska@biol.uni.lodz.pl (M.W.); rafal.szelenberger@biol.uni.lodz.pl (R.S.); marcin.niemcewicz@biol.uni.lodz.pl (M.N.); 2 Faculty of Advanced Technologies and Chemistry, Military University of Technology, 2 gen. S. Kaliskiego St., 00-908 Warsaw, Poland; piotr.harmata@wat.edu.pl; 3 Department of Pharmaceutical Microbiology and Biochemistry, Medical University of Lodz, 92-215 Lodz, Poland; tomasz.poplawski@umed.lodz.pl

**Keywords:** Ochratoxin A, liver, hepatotoxicity, gluconeogenesis, oxidative stress, apoptosis, prevention

## Abstract

Ochratoxin A (OTA) is considered as the most toxic of the other ochratoxins synthesized by various fungal species belonging to the *Aspergillus* and *Penicillium* families. OTA commonly contaminates food and beverages, resulting in animal and human health issues. The toxicity of OTA is known to cause liver damage and is still being researched. However, current findings do not provide clear insights into the toxin mechanism of action. The current studies focusing on the use of potentially protective compounds against the effects of the toxin are insufficient as they are mainly conducted on animals. Further research is required to fill the existing gaps in both fields (namely the exact OTA molecular mechanism and the prevention of its toxicity in the human liver). This review article is a summary of the so far obtained results of studies focusing on the OTA hepatotoxicity, its mode of action, and the known approaches of liver cells protection, which may be the base for expanding other research in near future.

## 1. Introduction

Ochratoxins are secondary metabolites produced by *Aspergillus* and *Penicillium* fungi. Among them, it can be distinguished into ochratoxin A (OTA), its methyl ester, ochratoxin B (OTB) and its methyl and ethyl esters, ochratoxin C (OTC), 4-hydroxyochratoxin A (4-OH OTA), and ochratoxin α (OTα). OTA is known as the most toxic [1] and was originally isolated from *Aspergillus ochraceus* Wilh. [1,2].

OTA can be produced in different climates depending on fungi species. Moderate and cold climate are mainly associated with *Penicillium verrucosum*, while warm and tropical with *Aspergillus ochraceus* [1]. The study performed by Wawrzyniak and Waśkiewicz showed that *P. verrucosum* could grow on various cereals at temperatures ranged from 10 °C up to 30 °C. However, the optimal temperature for OTA production was 20 °C and the highest OTA level was identified in rice [3]. *P. viridicatum* can more efficiently produce OTA in wheat and barley with a_w_ between 0.90 and 0.93. Lower a_w_ (0.85–0.86) resulted in decreased production of the toxin [4]. The best temperatures for the growth of three strains *A*. *carbonarius*, *A*. *niger*, and *A*. *ochraceus* in different culture media are 30 °C, above 30 °C, and 25 °C to 30 °C, respectively. *A*. *niger* is able to grow even at 41 °C, while this temperature is too high for *A*. *carbonarius* and *A*. *ochraceus.* In addition, none of them is able to grow at the temperature of 8 °C in any media. Moreover, *A*. *niger* occurred to be the most xerophilic among them [5]. The conditions under which *A*. *ochraceus* and *A*. *carbonarius* could produce the highest levels of OTA in coffee beans are temperatures between 25 and 30 °C with a_w_ between 0.97 and 0.99 and temperatures 22–32 °C with a_w_ 0.95–0.99, respectively [6]. On maize kernels *A*. *niger* could produce OTA in temperatures ranging from 15 to 40 °C, while *A*. *carbonarius* in temperatures ranging from 15 to 35 °C. For both strains, the highest OTA level was produced at 15 °C but it was not significantly different than the level produced at 20 °C for *A*. *carbonarius*. Comparing the influence of a_w_ at 0.92, OTA concentration was significantly lower for *A*. *niger* than at 0.96 and 0.98. For *A*. *carbonarius* the best a_w_ for OTA production was 0.98 [7].

OTA occurs in many food products and beverages as reported in many studies. It contaminates wheat [8], flour [9], rye [10], maize [11], rice [12,13], and coffee [14,15]. Some studies described the OTA presence in baby food products [16,17,18]. This toxin occurs in dry fruits [19], spices [20,21,22], herbs [21], and chili sauce [23,24]. Other OTA-containing products are wine [25] and beer [26]. OTA can also occur in animal-derived products due to consumption of contaminated feeds [27]. OTA contaminates salami [28,29,30], chicken meat, eggs [31], milk [32], and cheese [33]. Some studies reported the OTA presence in human blood [34], milk [35], and urine [36,37,38].

OTA possesses an embryotoxic, teratogenic, genotoxic, neurotoxic, nephrotoxic, and immunosuppressive features [39]. According to the International Agency for Research on Cancer, OTA was classified as a potential human carcinogen under group 2B, thus highlighting its multidirectional and harmful character [40]. To prevent health risks associated with OTA toxic effects, the maximum levels have been set in various food products by The European Commission. In dry vine fruit, is set to 8.0 µg/kg, in dried herbs to 10.0 µg/kg, in unprocessed cereal grains to 5.0 µg/kg, in instant coffee to 5.0 µg/kg, in wine to 2.0 µg/kg and in children and babies food products to 0.50 µg/kg [41].

Research studies published in recent years indicate the OTA toxic effect on human liver. For this reason, it was decided to collect information concerning the toxin impact on that organ, its mode of action, and the potential of reducing the hazardous effect.

## 2. General Information

### 2.1. Chemical Structure and Physical and Chemical Properties

**Figure 1:**
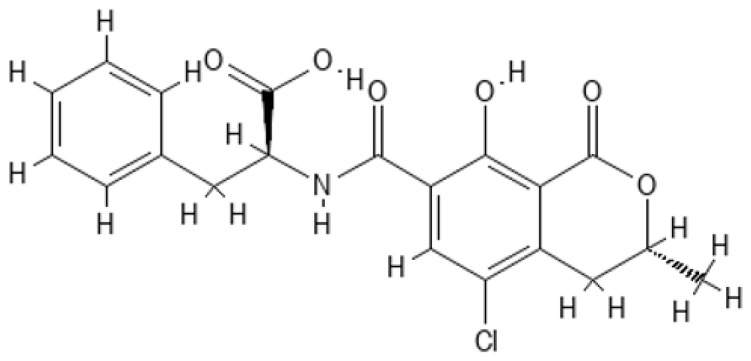
OTA schematic chemical structure (structure prepared using PubChem Sketcher [44]. Available online: https://pubchem.ncbi.nlm.nih.gov//edit3/index.html (accessed on 6 August 2023)).

OTA is a compound consisting of phenylalanine and dihydro-isocumarin and these two molecules are bound together by an amide bond (Figure 1) [42]. The molar mass of toxin is 403.8 g/mol [42,43]. OTA possess a colorless or white crystalline structure. It is a weak organic acid and its pKa value is 7.1. In acid and neutral pH, is soluble in polar organic solvents, moderately in water, and insoluble in petroleum ethers and saturated hydrocarbons. OTA is soluble in all alkaline solutions. This toxin keeps strong fluorescence which also depends on pH. In acid solutions, can emit green fluorescence and blue fluorescence in alkaline solutions. OTA is a stable compound that can withstand high temperatures and acidity [43]. OTA exists in three different forms: non-ionic, monoanionic (OTA^−^), and dianionic (OTA^2−^) [42].

### 2.2. Biosynthesis

OTA biosynthesis requires multiple genes as it is a complex process. Studies conducted on various OTA-producing strains show similarities in toxin biosynthesis.

The presence of polyketide synthase (PKS) is crucial for OTA production [45,46] and is encoded by the *An15g07920* gene [46]. The disruption of the gene resulted in a lost capacity of OTA biosynthesis in *A*. *niger* [46]. The study performed on *A*. *westerdijkiae* NRL 3174 shows that *aoks1* is a PKS gene required in the OTA biosynthesis [47]. Färber et al. used the *P. nordicum* BFE487 strain in their study and found several fragments with homology to enzymes known as potentially involved in OTA biosynthesis such as PKS, halogenase, phenylalanine t-RNA synthetase, methylase, and ABC transporter genes [48]. Two out of three *P. nordicum* strains could express *otapksPN* and *otanpsPN* genes, which are involved in OTA production in the presence of NaCl [49]. Ferrara et al. performed a study on *A*. *carbonarius* ITEM 5010. The researchers reported an upregulation of the cyclase gene (*otaY*). After its deletion OTA, was not detected in culture extract after seven days of incubation in the dark. The toxin was found in a wild type. However, the lack of cyclase gene did not prevent or significantly alter the expressions of *otaA*, *otaB*, *otaC*, *otaD* and *otaR1* related to OTA biosynthesis [50]. The results of the research on *A*. *niger* performed by Wei et al., showed a valid role of the *AnAzf1* gene. After its removal, OTA production was completely blocked and cluster of genes such as: *p450*, *nrps*, *hal*, and *bzip* was repressed at the transcriptional level [51]. The *AcOTAbZIP* gene is directly related to the expression of four other genes: *AcOTApks*, *AcOTAp450*, *AcOTAnrps*, and *AcOTAhal*. As reported by Gerin et al., *A*. *carbonarius* could not synthesize OTA after deleting the *AcOTAbZIP* gene [52]. The study performed by Gallo et al., shows that inactivation of *A*. *carbonarius AcOTAnrps* gene led to complete inhibition of OTA biosynthesis [53]. Two transcriptional factors are involved in the biosynthesis of OTA – *veA* [54,55] and *laeA* [45,54]. After the removal of both transcriptional factors from *A*. *carbonarius* the production of OTA was significantly decreased. Under the dark conditions for *ΔveA* transformant production of the toxin was decreased by 99.4% and for *ΔlaeA* transformant was decreased by 97%. Under light conditions, OTA production was decreased by 89% and 68.5%, respectively [54]. The *veA* gene acts as a positive regulator of OTA biosynthesis in *A*. *niger* in both light and dark conditions [55]. The *ΔAclaeA* of *A*. *carbonarius* strain produced much less OTA and had lower relative expression of OTA cluster genes such as: *pks*, *nrps*, *bZIP*, *p450* and *hal* comparing to wild type. The lack of *Acpks* down-regulated the transcript levels of *laeA* and the same cluster genes (*nrps*, *bZIP*, *p450* and *hal*) as in the case of *ΔAclaeA*. Glucose oxidase (GOX) was reported as another important element in the biosynthesis of OTA. The *ΔAcgox* strain had reduced OTA production in comparison to the wild type. Moreover, in this strain the expression of *laeA* was decreased suggesting their dependency [45]. These results indicate a close relationship among individual genes, which only underlines their importance during OTA biosynthesis. This is attributed to the phenomenon, where the deletion of specific genes may results in the suppression of OTA synthesis. The residual genes are insufficient for OTA production, indicating that their mere presence does not guarantee the fungi’s ability to biosynthesize the toxin. This is substantiated by the observation that strains lacking any genetic alterations retained the capability to produce the toxin. The genetic modifications executed in the studies on OTA-producing strains culminated in the loss of their toxin-producing capacity.

### 2.3. Toxicokinetics

Absorption of OTA varies among different species. In the study conducted by Hagelberg et al., the OTA concentration in plasma after its administration showed that relative bioavailability in pigs was about 60%, in mice reached 97%, in rats achieved 44% [56] and in human body attained 93% [57]. These differences show that establishing a specific animal model for OTA studies is necessary.

Once OTA enters the organism, its distribution to tissues is species-dependent. Several factors can influence this phenomenon, such as: the toxin quantity, the way of ingestion, overall health status, and diet composition. The major organs susceptible to the distribution and OTA accumulation include: kidneys, liver, skeletal muscles, and even the brain [1,58]. Interestingly, Zimmerli et al. showed that OTA was also found in fetal serum, thus indicating a potential role of active placental transport [59]. Moreover, the kidneys and liver exhibit the highest sensitivity to OTA, likely due to their transporting mechanisms. Currently, scientists indicate the crucial role of organic anion transporters (OATs) that facilitate the OTA transport through the basolateral membrane of the proximal tubule [1]. In Jung et al. study, two different human multi-specific OATs were isolated from the kidney—hOAT1 and hOAT3. Besides different localization of those OATs, both were demonstrated to be involved in the OTA transport [60]. While basolateral OATs primarily facilitate OTA uptake from the bloodstream into kidney tubule cells, the apical OAT4 transporter may contribute to OTA’s reabsorption in urine, leading to its accumulation in tubule kidney cells [42].

It is suggested that OTA departs the kidney cells across the luminal membrane by passive transport. This assumption was discovered by Bahnemann et al. study on the toad kidney model. Authors showed that facilitated diffusion was supported by transport mechanism vulnerable to probenecid—a specific inhibitor of the ATP Binding Cassette Subfamily C (ABCC) [61]. This hypothesis was also confirmed by Leier et al. In their study, a recombinant human MRP2 (ABCC2) was shown to be involved in the apical efflux pump in OTA excretion [62].

As mentioned previously, the second organ susceptible to OTA is the liver. Hepatocytes absorb significant portion of OTA and then excrete it into the bile. In the study conducted by Kontaxi et al., a radiolabeled [3H]OTA was used to identify hepatocellular uptake in isolated rat hepatocytes. Obtained results showed, that OTA transport was saturation, temperature, and energy dependent. Furthermore, the OTA uptake was reduced by different bile acids, sulfobromophthalein, and the thrombin inhibitor CRC 220m which are known to be a substrate for organic anion-transporting polypeptides (OATPs) [63]. The important role of OATPs in OTA distribution in the liver was also confirmed in the Wang et al. study, in which human OATP1B3 protein affected OTA pharmacokinetics in vivo in humanized mice model [64]. At the current state of the knowledge, OATPs are considered as the main OTA regulators in distribution process in the liver. However, the number of studies focused on the liver OTA toxicokinetics is strongly limited. It would be beneficial to examine the role of OATPs in OTA distribution in human cells, for the purpose of better understanding the molecular processes and its hepatotoxicity backgrounds.

### 2.4. Metabolism in the Liver

The liver microsomes are involved in OTA metabolism. Studies have shown that, cytochrome P450 (CYP450) is responsible for the formation of hydroxylated forms of OTA such as (4S)-4-OH OTA and (4R)-4-OH OTA in human, pig, rat and rabbit [65,66], and 10-hydroxyochratoxin A in rabbit [66]. Omar et al., studied rats’ liver microsomes for their ability to metabolize OTA with different CYP450 inducers. 3-methylcolcanthrene (3MC), isosafrole (ISF), phenobarbital (PB), dexamethasone (DXM), and clofibrate (CLF) led to significant increase in the (4R)-4-OH OTA formation. In the case of the formation of (4S)-4-OH OTA caused by these inducers, the increase was smaller. Isoniazid (INH) inducer acted differently. It increased better the level of (4S)-4-OH OTA [67]. In rats’ liver microsomes, the (4R)-4-OH OTA formation reached the highest level with CYP450 II-a. Type II-b produced less metabolite, while type II-c the least. The activity of type I CYP450was not as efficient in comparison to type II. In the case of formation of (4S)-4-OH OTA, its level was the highest with type I-c [68]. Previous research results from 2002 showed, three different metabolites formed in cultured rat primary hepatocytes. Except for hydroxylated OTA, the researchers found acyl hexose conjugate and acyl pentose conjugate [69]. Yang et al. also conducted a study on rats’, chickens’, swine’s, goats’, cows’ and humans’ liver microsomes. The researchers found six different OTA metabolites. Four of them were 4(S)-OH-OTA, 4(R)-OH-OTA, 7′-OH-OTA and OTB, while the other two were considered most likely as 5′-OH-OTA and 9′-OH-OTA [70]. The toxicity of OTA metabolites is lower in comparison to OTA itself [71].

The results of the studies indicate the formation of similar OTA metabolites. Researchers agree on several types that occur in both animals and humans, suggesting resemblances in livers OTA metabolism in various organisms. However, these studies do not show the formation of acyl hexose and acyl pentose conjugates in the human but focusing on rat model, which may indicate the existing difference among animals and humans. Moreover, in the case of 10-hydroxyochratoxin A its formation is characteristic for rabbit. (4R)-4-OH OTA and (4S)-4-OH OTA are present in all mentioned studies, suggesting that, these are major metabolites generated in the livers.

## 3. The Impact of OTA on Hepatocytes and Hepatotoxicity

A broad spectrum of OTA’s activity was shown in many studies. Most of them focused on multiorgan toxicity, with an emphasis on kidneys [72]. However, an insufficient number of studies were focused on the hepatotoxic role of OTA and the hepatoprotective features of various chemical substances. It indicates the necessity to fill the existing gap, especially since the liver is strongly involved in the mycotoxins biotransformation and detoxification [73].

Gayathri et al. demonstrated that OTA induced apoptosis of the HepG2 liver cell line, chromatin fragmentation, generate reactive oxygen species (ROS) production, and causes DNA damage. Furthermore, based on the results from the mitochondrial trans-membrane potential and the concentration of caspases and apoptotic-associated proteins (Bax and Poly(ADP-ribose) Polymerase (PARP)), the authors suggested that, the apoptotic cell death was mediated mainly by mitochondria [73]. Similar results, according to the cell viability, apoptosis, and oxidative stress, were also presented by Shin et al. What is more, the authors evaluated the activity of the Nrf-ARE signaling pathway, which is associated with the defensive mechanism against oxidative stress. Further, more detailed studies using siRNA transfection for silencing Ahr, the transcription factor responsible for the toxic substances oxidation and Nrf2, the key regulator of the antioxidant defense system, showed that DNA damage and apoptosis were induced as the result of ROS generation by cytochrome (CYP) enzymes, and as a result of increased mRNA expression of 3 and 9 caspase. Authors suggest that occurred damages may be linked with impair function of defensive system responsible for lowering oxidative stress, thus emphasizing ROS generation as a crucial factor of OTA-induced cytotoxicity. Furthermore, gene expression and protein concentration of CYP450 (CYP1A1, CYP1A2) and antioxidant enzymes (heme oxygenase-1, HO-1, and glutamate-cysteine ligase, GCL) in in vivo mice model was consistent with the results obtained in in vitro studies [74].

In study conducted by Qi et al., the miRNA expression profile in the livers of rats treated with OTA was examined. Results showed that, in the high-dose group, 47 miRNAs were upregulated, and 26 were downregulated in comparison to control livers cells. In the medial-dose group, 59 miRNAs were upregulated, and 47 were downregulated. Overlapping results shown 29 upregulated and 14 downregulated miRNAs. The differences in the expression were also studied in the mRNA profile in order to identify the mechanisms and pathways influenced by OTA treatment. In the high-dose group, OTA treatment was associated with circulatory, endocrine, digestive, and excretory systems, signal transduction, and lipid metabolism, which are solely linked with different body systems. On the other hand, in the medial-dose group, the differences were linked with amino acid, carbohydrates, cofactor and vitamin metabolism, and signaling molecules and overall interaction, which were mainly related to energy metabolism. The obtained results showed that, the doses of OTA may induce different toxicity mechanisms in the liver. Furthermore, to verify the identified abnormalities in miRNA and mRNA expression profile, the authors performed two-dimensional electrophoresis, to evaluate the proteomic alterations. Obtained results showed that, the expressions of 61 proteins were significantly different in the high-dose and control group. Bioinformatic analysis indicated various proteins expressions associated with cysteine and methionine metabolism, peroxisome proliferator-activated receptor (PPAR) signaling, arginine, and proline metabolism, primary bile acid biosynthesis, and metabolism of xenobiotics by CYP450 [75].

Wang et al. performed a study, in which ducklings were treated with oral gavage of OTA for 2 weeks. Results showed that, OTA administration induced changes in the intestinal microbiota composition, and caused the accumulation of lipopolysaccharides (LPS) and liver inflammation, which was confirmed by molecular techniques and histological analysis. Ducks livers had augmented expression of TLR-4, Myd88, and p-p65 (at the mRNA and protein level), which are the known factors that stimulate NF-ĸB pathway [76,77].

## 4. The Mechanisms Responsible for Liver Damage

As mentioned in the Section 3, OTA was shown to have direct and indirect effects on hepatotoxicity and overall liver damage, in both in vitro and in vivo studies. However, the molecular mechanism of action is not fully discovered. Thus, several different pathways may be responsible for liver damage.

### 4.1. Gluconeogenesis

Gluconeogenesis is a metabolic pathway, mainly occurring in the liver and kidneys, in which noncarbohydrate metabolic substrates are transformed into glucose. Gluconeogenesis is performed serially in eleven enzyme-catalyzed reactions and its regulation is dependent on the amount of pyruvate carboxylase, phosphoenolpyruvate carboxykinase (PEPCK), fructose-1,6-bisphosphatase, and glucose-6-phosphatase [78,79]. The key regulatory step in gluconeogenesis is associated with the conversion of oxaloacetate to phosphoenolpyruvate by PEPCK, which is known as a high-energy reaction [78]. In study performed by She et al., mice with the deletion of the *pck* gene, which encodes the PEPCK, died within three days of birth. Furthermore, one-day-old *pck*-null mice had strongly elevated hypoglycemic. Results of this study showed, that PEPCK is essential for life and its disorders may lead to impaired gluconeogenesis, thus emphasizing its crucial role in this metabolic pathway [79]. Research performed in 1981 and 1986 showed that orally OTA administration to rats showed 50% reduced activity of renal PEPCK. In the same study, the concentration of the hepatic PEPCK was not changed, which may indicate that gluconeogenesis in the liver is unaffected. However, very interesting results were obtained after binding OTA with albumin. Administration of covalently bind OTA and albumin, showed 34% decrease in hepatic PEPCK activity [80]. A study conducted by Il’ichev et al. showed, that OTA has at least two unique sites that enable its binding to human serum albumin (HSA) predominantly as dianion [81]. This phenomenon may be crucial for prolonged life span and long-term activity of OTA but also elevated passive absorption and perfusion of organs [82]. Thus, OTA may influence the gluconeogenesis in liver by binding with HSA.

**Figure 2:**
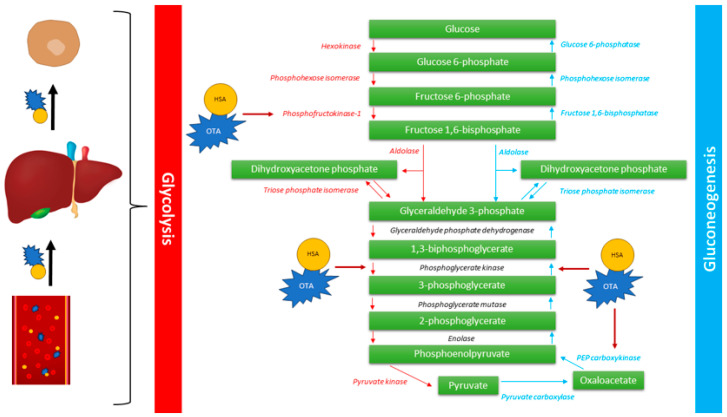
Hypothetical mechanisms of OTA-induced impaired glycolysis and gluconeogenesis. OTA binds HSA in the bloodstream, which increases the perfusion into the liver. Derived into hepatocytes, OTA influence PEPCK, phosphofructokinase-1, and phosphoglycerate kinase.

In the study performed by Hundhausen et al., OTA-treated Hep2G cells were used in the screening analysis of the mRNA profile by microarrays. Results showed that besides the *pck2* gene, *pfkl* and *pgk1* were also downregulated. Phosphofructokinase, which is encoded by the *PFKL* gene, is crucial liver-type enzyme that catalyzes the conversion of D-fructose 6-phosphate to D-fructose 1,6-bisphosphate, which is a main step in glycolysis. Phosphoglycerate kinase 1, encoded by the *PFGK1* gene, is an enzyme that catalyzes the conversion of 1,3-diphosphoglycerate to 3-phosphoglycerate in glycolysis [83]. At the current state of the art, no studies are focusing on OTA role in hepatic glycolysis. However, molecular studies showed the necessity for the detailed examination of all hepatic metabolic pathways, including gluconeogenesis and glycolysis, especially in both free OTA and HSA-OTA form (Figure 2).

### 4.2. PPAR Signaling Pathway

**Figure 3:**
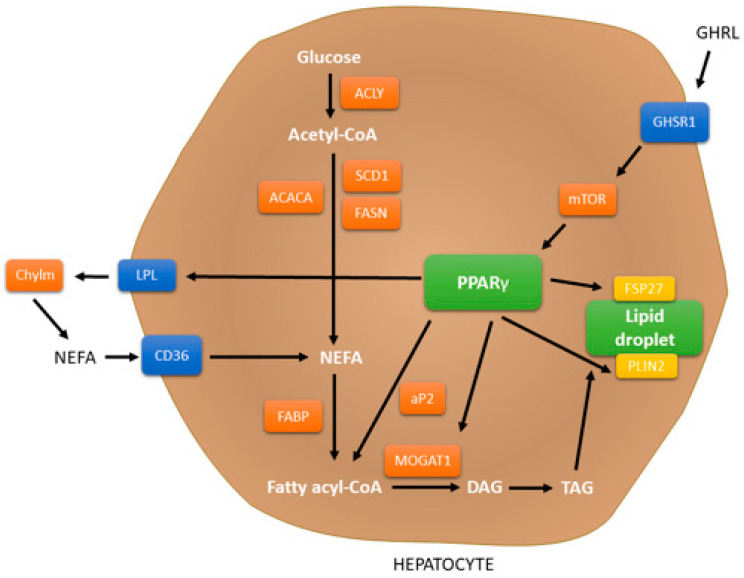
Signaling pathways of PPARγ in hepatocytes. Activated PPARγ regulates the NEFA uptake target genes such as *CD36* and *LPL*, *FABP* for lipid metabolism, *aP2* for transporting NEFA and fatty acyl-CoA, *MOGAT1* which is responsible for the synthesis of DAG, *PLIN2* and *FSP27* for the accumulation of the lipid droplet, and *ACLY*, *ACACA*, *SCD1* and *FASN* responsible for glucose transformation. Abbreviations: *ACACA*—Acetyl-CoA Carboxylase, *ACLY*—ATP Citrase Lyase, *aP2*—Adipocyte Protein 2, *CD36*—Cluster of Differentiation 36, *Chylm*—chylomicron remnants, *DAG* —Diacylglycerol, *FABP*—Fatty Acid Binding Protein, *FASN*—Fatty Acid Synthase, *FSP27*—Fat specific Protein 27, *GHRL*—Ghrelin, *GHSR1*—Ghrelin receptor 1, *LPL*—Lipoprotein lipase, *MOGAT1*—monoacylglycerol 0-acyltransferase 1, *mTOR*—mammalian target of rapamycin, *NEFA*—non-esterified fatty acid, *PLIN2*—Perilipin 2, *PPARγ*—Peroxisome proliferator-activated receptor gamma, *SCD1*—Stearoyl-CoA Desaturase, *TAG*—triacylglycerol.

PPAR belongs to the nuclear receptor superfamily of ligand-inducible transcription factors. The three main isoforms are presented in mammals: PPARα (also known as NR1C1), which was first identified in 1990, PPARβ/δ (NR1C2), and PPARγ (NR1C3). PPARs are able to control the expression of genes associated with inflammation, lipid metabolism, adipogenesis, and metabolic homeostasis. PPARα is mainly expressed in liver, brown adipose tissue, and heart, and is linked with the oxidation pathways of fatty acid. PPARβ/δ shows parallel functions as PPARα is also expressed in the liver and heart [84,85]. PPARγ may be expressed in three isoforms, based on the splice variants of mRNA. PPARγ1 is widely expressed, PPARγ2 is mainly expressed in liver and adipose tissue, whereas PPARγ3 is mostly expressed in adipose tissue and colon. Crucial role of PPARγ is associated with the fatty acid uptake, lipogenesis, insulin sensitivity and formulation of lipid droplets [86]. The contribution of PPARγ in the liver was presented in Figure 3 [85,87].

**Figure 4:**
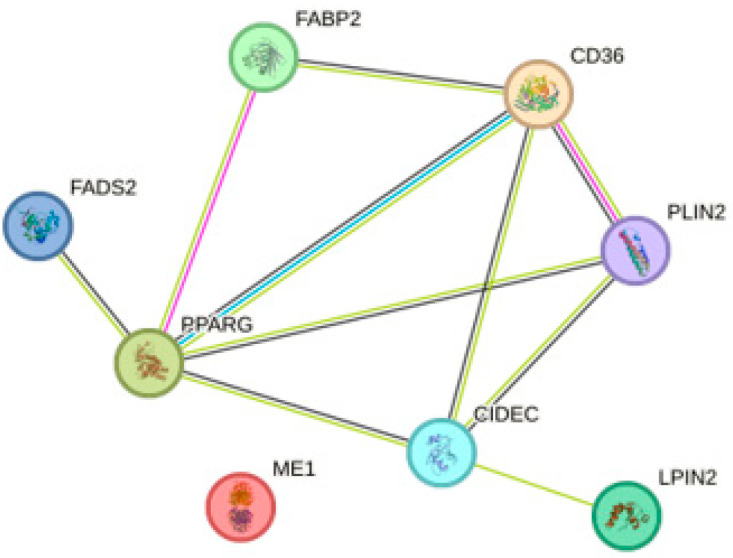
The protein-protein interaction (PPI) analysis associated with PPARγ pathway. PPIs were determined using the STRING database [89].

In the study conducted by Zhen et al., OTA administration was shown to impact PPARγ. Firstly, the authors showed that OTA caused the accumulation of lipid droplets in Hep2G cells and isolated primary hepatocytes in a dose-dependent manner. Secondly, the effect of OTA was also confirmed in vivo. After 12 weeks of OTA treatments, mice showed a sign of early steatosis, which confirms the lipid accumulation in hepatocytes. Further performed bioinformatic analysis showed predominantly altered mRNA genes expression associated with the PPAR signaling pathway. Results obtained from next-generation sequencing and qPCR of primary hepatocytes showed upregulation of *Fabp2*, *Cd36* (associated with lipid uptake), *Me1*, *Lpin2*, *Fads2* (associated with lipogenesis), *Plin2* and *Fsp27* (associated with lipid droplet assembly) genes. Similar results were obtained for the HepG2 cell line, however, the expression of *FADS2* was not statistically significant. Furthermore, the concentration of PPARγ was also increased. To verify the connection between PPARγ activity and elevated steatosis, authors used PPARγ antagonist – GW9662. Results showed that suppressed PPARγ prevented lipid accumulation in hepatocytes. The possible explanation of this phenomenon was linked with post-translational ubiquitination, which was experimentally confirmed. It is also worth mentioning that both Cluster of Differentiation 36 (CD36) and Fatty Acid Binding Protein 2 (FABP2) had a crucial role in the lipid accumulation, which was shown after the gene silencing experiment [88]. Based on these data, protein-protein interaction (PPI) analysis was performed. It showed that FADS2 and CD36 do not manifest mutual interaction. However, they both interact with PPARγ. Thus, this suggests that lipid accumulation after OTA treatment may be associated by more than one factor (Figure 4).

Upregulation of genes associated with the PPAR signaling pathway (according to KEGG pathways) were also observed in the chicken hepatocellular carcinoma cell - LMH (which was widely used to evaluate the toxic mechanisms of mycotoxins) after exposure to OTA [90].

Deregulation in the PPAR signaling pathway was also demonstrated in the Qi et al. [75] study, in which several miRNA, mRNA, and protein levels associated with the PPAR signaling pathway were altered in the rat liver after OTA treatment. Based on the obtained results of cross-omic analysis, authors suggested that OTA treatment influences 5 main pathways (cysteine and methionine metabolism, PPAR signaling, primary bile acid biosynthesis, arginine and proline metabolism, and metabolism of xenobiotics) and induced early hepatotoxicity [75].

### 4.3. Oxidative Stress

OTA has been implicated in wide range of harmful, toxicological effects, including DNA damage. Currently, the exact mechanisms of action are not fully understood, but it is hypothesized that oxidative and nitrative stress may play a significant role in genotoxicity and liver disorders. Several distinct mechanisms involved in the oxidation and reduction processes induced by OTA have been reported. One of these mechanisms involves the capacity to chelate Fe^3+^ ions, facilitating their easier reduction to Fe^2+^ ions, which in the presence of oxygen initiate the lipid peroxidation process. This mechanism has been proposed in reconstituted system comprising phospholipid vesicles, Fe^3+^ ions, and the flavoprotein NADPH-cytochrome P450 [91]. Other research showed, that OTA induces oxidative damage through the generation of hydroxyl radicals. Furthermore, the reaction conducted in the presence of oxygen, NADPH, and cellular microsomes did not require the presence of iron ions [92,93]. In the study conducted by Shin et al., where Hep2G cell culture was treated with OTA, the generation of ROS increased in a dose-dependent manner. Moreover, the concentration of malondialdehyde (MDA), which is a lipid peroxidation product, and nitric oxide (NO), which is released in response to oxidative stress, was also significantly augmented. Further, the concentration of glutathione (GSH), which is well-known antioxidant, was significantly decreased [74]. The diminished concentration of GSH was in line with the observations of Atroshi et al. [94] and Gross-Steinmeyer et al. [69]. The augmented generation of ROS (including O_2_^●−^, HOO^●^, and OH^●^) was also observed in the Guerra et al. study [95]. What is more, the generation of oxidative stress was also shown in the Domijan et al. study, in which OTA caused protein carbonylation in rat’s liver [96]. The reported findings suggest that the mechanism led to cell damage, including DNA damage, arises not only through the generation of ROS but also through the inhibition of the antioxidant system. One of the most frequently postulated hypotheses related to the inhibition of the defensive activity against oxidative stress is the ability of OTA to reduce the expression of genes regulated in the Nrf2-ARE pathway [97]. However, the results remain contradictory to each other. Shin et al. showed that Nrf2 expression (at the mRNA and protein level) increases with extended OTA concentration. What is more, augmented expression of Nrf2 was shown in the nucleus, not in the cytosol. Thus indicating that OTA may led to the translocation of Nrf2 from the cytoplasm into the nucleus [74]. On the other hand, in the study conducted by Cavin et al. primary rat hepatocytes after OTA treatment showed decreased expression of proteins regulated by Nrf2 pathways, including NQO1, GSTP1, GSTA5, GSTM1, AKR7A1, and GCLC. Furthermore, the authors demonstrated that, OTA inhibits Nrf2-DNA binding in time- and dose-dependent manner in primary hepatocytes. Additionally, in the rat liver cell line RL-34, OTA resulted in the suppression of Nrf2 activity after OTA treatment [98]. Similar findings were discovered in the Zhang et al. study, in which OTA administration caused 58.1% downregulation (*p* < 0.05) in the mRNA expression and 9% in the protein expression (*p* < 0.05) of Nrf2 in rabbit’s liver [99]. These results may be associated with the ability of OTA to reduce protein synthesis in cells [95]. According to the data presented by Cavin et al., downregulated proteins involved in the Nrf2 pathway in the KEGG Pathways [100] and DAVID Bioinformatic Resources were analyzed [101,102]. The analysis showed that reduced expression of NQO1, GSTP1, GSTA5, GSTM1, AKR7A1, and GCLC may also lead to decrease the level of N-acetyl-L-cysteine (NAC). NAC is GSH precursor, which increases its concentration, elevates NO levels, and provides overall anti-inflammatory activity [103]. In the study conducted by Sueck et al., the conjugated OTA-NAC metabolite was observed in human urine [104]. Furthermore, the protective effect of NAC against DNA damage, cell viability, and ROS generation (NAC caused elevated mitochondrial membrane potential and sodium dismutase activity) was shown in the human embryonic kidney cells HEK-293 [105]. Thus indicating, that liver damage may be associated not only with the Nrf2-ARE pathway but also with the GSH metabolism.

The discrepancy among studies may result from the presence of various differentiating factors, such as cell line type, origin, exposure time, applied dosage, as well as the form of OTA administration. Therefore, a greater number of studies focusing on determining the activity of the Nrf2-ARE pathway and GSH metabolism in liver cells are necessary.

The metabolism of OTA presents similarities and analogies with the chlorophenol toxin—pentachlorophenol (PCP). The use of PCP in research on OTA is relatively common, particularly due to the fact that OTA is referred as natural chlorophenol mycotoxin [106,107]. In the case of PCP, oxidative dechlorination performed in the presence of cytochrome P450 causes the formation of electrophilic tetrachlorobenzoquinone (TCBQ). TCBQ possesses the ability to react covalently with sulfhydryl groups [108] and DNA bases [109] to form benzetheno-type adducts. Since oxidative stress can be likened to self-propelled machine, the generation of O_2_^•−^ will result in the formation of Fe^2+^ and H_2_O_2_, which subsequently, through the Fenton reaction, will produce HO^•^, leading to oxidative DNA damage [106,110]. In the case of OTA bioactivation, Pfohl-Leszkowicz, and Manderville suggested the analogy of activated pathways with PCP [106]. OTA in the presence of cytochrome P450 forms an electrophilic quinone (OTQ) during the oxidative dechlorination pathway [111]. Further, OTQ reacts with GSH and with amidst ascorbate, forms hydroquinone (OTHQ) [112], thus enhancing the oxidative stress and resulting in DNA damage (which was shown in the Kamp et al. study) [113].

The information gathered in this subsection requires however more detailed investigations. The majority of conducted studies were performed on human kidney cell lines or the kidneys of various animals. However, it should not be forgotten that, the uncertainties indicated in our hypotheses may also involve the liver. Given its significant role in the metabolism of various chemical compounds, the presence of the same molecular pathways, as well as the highest concentration of CYP450, which plays a crucial role in OTA metabolism. Conducting more detailed studies on liver cells could provide immense implications in determining the molecular mechanism of hepatotoxicity, and consequently, identifying hepatoprotective agents that could potentially save the health and lives of many individuals.

### 4.4. Apoptosis

Liver damage may also be caused due to induced apoptosis, the programmed cell death. Atroshi et al. showed that, the mice livers after two weeks of OTA administration had an 8.3-fold augmented amount of apoptotic bodies compared to the not-treated, control livers (*p* < 0.01) [94]. In the Chopra et al. study, the OTA-induced apoptosis of primary rat hepatocytes was investigated. Authors showed, that of 84 studied genes, 16 were upregulated and consisted of both: pro- and anti-apoptotic (i.e., *tp53*, *bad*, *apafl*, *bcl2a1*, *mcl-1*, *bnip2*, *bnip3*, *birc5*, *caspase 7*). It was suggested that OTA apoptosis might be associated with DNA damage and subsequent activation of p53-target genes, especially *bad* and *bax*, which are p53-target genes also generated on the protein level. Furthermore, obtained results showed that OTA-treated hepatocytes had a significantly elevated rate of nuclear apoptotic events, based on the DAPI staining. Moreover, the activity of caspase-3, -7, -8, and -9 was provoked by OTA treatment. Thus, the authors suggested that observed disturbances (increased activity of caspase-3 and -9) may be related to oxidative stress and damaged mitochondria in the intrinsic pathway [114]. Similar results were obtained in the Shin et al. and Bouaziz et al. study [74,115]. Besides the activation of extrinsic and intrinsic apoptotic pathways, authors found that OTA causes only partially caspase-dependent apoptosis. Obtained results showed, that OTA did not induce ICAD-L (long isoform of inhibitor of caspase-activated DNase) cleavage, which may suggest the presence of other factors linked with internucleosomal DNA fragmentation. Furthermore, inhibition of caspase activity did not fully affect the induced apoptosis. Thus, it suggests that a crucial role may be related to endonuclease G, which was found only in nuclei of OTA-treated hepatocytes [114]. In the study performed by Bouaziz et al., the signaling pathway of OTA-induced apoptosis was presented. Authors showed that OTA in Hep2G cells provoke DNA damages that lead to caspase-mediated mitochondria-dependent apoptosis. In the suggested pathway, Bax relocates to the mitochondrial outer membrane, resulting in the opening the permeability transition pore complex (PTPC) and loss of mitochondrial membrane potential, which leads to the release of cytochrome c and activation of executive caspases. Moreover, OTA initiates a p53-dependent apoptotic process with diminished level of ROS generation, as a result of the mitochondrial modifications [115].

In the studies focused on the role of OTA on hepatocyte apoptosis, it was also shown that OTA stimulates secretion of TNFα, which is crucial ligand for extrinsic pathway [116]. However, there is not enough data to analyze the molecular mechanism of activation of this apoptotic pathway in hepatocytes. Thus, more detailed studies are necessary.

## 5. Prevention

Apart from studies that highlight the OTA hepatotoxicity, there are also reports that emphasize the protective properties of certain substances which can reduce the toxin harmful effects on the liver. Unfortunately, due to the fact that the experiments were conducted on animals models, only a few of them focus on the use of human cells, thus leaving a gap in this field. Animals are used in biological research and medicine due to their anatomical and physiological similarities to humans, especially in the case of mammals. However, the results cannot be directly translated to humans [117]. Pound et al. [118] summarized methodological problems of animals experiments. The drug efficacy and toxicity can vary depending on the species and strains of animals due to differences in metabolic pathways and drug metabolites. Additionally, they identified issues such as: small experimental groups with limited power, simplistic statistical analysis, various models for inducing illness or injury with different level of similarity to the human condition, and uncertain relevance of drug dosing schedules and regimens to humans [118]. These are not all of the limitations while conducting research on animal model, but only emphasizes that studies performed on humans are needed.

### 5.1. Antioxidant Substances

#### 5.1.1. Vitamin E

A. Gayathri et al. reported that, vitamin E can reduce ROS levels and increases the viability of the HepG2 cell line as vitamin co-administration ensures protection against OTA-induced cytotoxicity [73]. The supplementation of this vitamin decreased the concentration of MDA in chicks [119]. By this way, vitamin E in some degree can counteract the toxic effects generated.

#### 5.1.2. Vitamin C

In the study performed by Shalaby Nile tilapia were fed with OTA contaminated food in two concentrations—400 and 600 µg. The results showed a significant decrease in the levels of liver total protein and aspartate aminotransferase (AST) activity. In the case of alanine aminotransferase (ALT), its activity was significantly higher in comparison to control. The administration of 500 µg of vitamin C improved these parameter by increasing both total protein levels and AST activity while decreasing the ALT action [120]. In the study it was observed an increase in the number of liver cells undergoing apoptosis in mice exposed to OTA. However, vitamin C administration proved to be protective and lowered the number of apoptotic hepatocytes [121].

#### 5.1.3. Quercetin

Quercetin has an anti-inflammatory effect in HepG2 cells as it weakens the ROS generation and NO accumulation, induces Nrf-2 nuclear translocation and expression and leads to suppression of NF-ĸB expression and retention in the cytoplasm. Pretreatment of cells with quercetin followed by OTA leads to lower expression of cyclooxygenase-2 (COX-2) [122]. It was proven that quercetin can mitigate immunotoxicity presumably by amelioration of generated by OTA oxidative stress.

Coadministration of honey or quercetin with OTA for 15 days significantly reduced DNA damage in mice’s livers. Both reduced the length of tails visible in the comet assay [123].

#### 5.1.4. Melatonin (MEL)

MEL, produced by the pineal gland, participates in various physiological functions. This compound was used by Sutken et al. in the study performed on rats. After four weeks, the OTA-treated rats expressed significantly higher levels of MDA and hydroxyproline (Hyp), while glutathione peroxidase (GPx) level was lower. The addition of MEL (OTA+MEL) markedly decreased levels of MDA and Hyp and increased GPx activity in the liver. It was observed that MEL at dosage of 10 mg/kg can reduce parenchymal and stromal degeneration in the liver [124]. MEL protects rats’ livers and supports antioxidant defense against OTA since it reduces levels of lipid peroxidation [125,126]. The addition of MEL in diet led to decreased level of MDA and increased levels of GPx and superoxide dismutase (SOD) in livers of rats [126,127]. As reported MEL improved the parameters of serum protein concentration and enzyme activities by increasing total protein and albumin levels, increasing creatinine kinase (CK) activity and decreasing alkaline phosphatase (ALP) and G-glutamyl transferase (G-GT) action [127]. In addition, this compound increased GSH levels, glutathione reductase (GR) and glutathione-S-transferase (GST) [126]. In that study, Cherry Valley ducklings were randomly grouped into four groups: control, OTA, MEL, and OTA+MEL. As reported by Xia et al. [128], the addition of MEL significantly reversed the dysbiosis of the intestinal microbiota. Moreover, the presence of MEL in the diet had a noticeable impact on reducing the LPS accumulation in the cecum induced by OTA. Therefore, MEL alleviated OTA-induced liver inflammation as is related to disruptions in intestinal microbiota and LPS accumulation. In addition, MEL also inhibited activation of the TLR4 signaling pathway as a result of decreasing mRNA expression and levels of TLR4, Myd88 an p-p65 proteins, ratio of p-IKBα/IKBα and decreased the secretion of liver cytokines such as IL-1β and TNF-α. Moreover, it was noticed that MEL decreased levels of serum AST, ALT, and ALP [128]. The results of the conducted research indicate the possibility of using MEL to protect the liver.

#### 5.1.5. Coenzyme Q_10_ (CoQ_10_)

The addition of CoQ_10_ worked similarly to MEL, since it significantly decreased MDA and Hyp levels and increased GPx activity. In comparison to the control, OTA+MEL results were closer, while OTA+CoQ_10_ were significantly different—higher for MDA and Hyp and lower for GPx. Moreover, CoQ_10_ treated group had decreased mononuclear cell inflammation and congestion in sinusoids [124].

### 5.2. Trace Elements

#### 5.2.1. Zinc

As reported, the zinc supplementation significantly reduced ROS production and decreased the activity of SOD. Zinc also helped to maintain the DNA integrity. The presence of OTA increased the mRNA expression of metallothionein1-A (MT1A), metallothionein2-A (MT2A), and superoxide dismutase 1 (SOD1), while zinc supplement additionally increased the expression of MT1A and MT2A but did not affect the expression of SOD1. Based on the findings, it is suggested that supplementing zinc can protect human liver cells from the negative effects of OTA, including oxidative stress, 8-hydroxy-2′-deoxyguanosine formation, DNA strand breaks, and DNA hypomethylation [129].

#### 5.2.2. Natural Form of Selenium–Selenomethionine

In vivo study showed protective effect of selenomethionine (SeMet) on rabbit liver. SeMet in concentration 0.4 mg/kg keeps positive impact as it regulates Nrf2/HO-1 expression by activating the Nrf2 signaling pathway and increasing HO-1 expression. Since this compound weakens the impact of OTA, it increases the antioxidant effect and protects the liver from oxidative damage [99].

### 5.3. Plants Extracts

#### 5.3.1. *Emblica officinalis* (amla)

*Emblica officinalis* known as amla has been used in the acient Indian Ayurveda as a potent rasayana. In the study from 2010 concerning protective influence of amla on OTA mouse liver cells, it is reported that after oral administration its aqueous extract led to significant improvement in lipid peroxidation caused by OTA in mouse liver as it decreased the levels of MDA [130].

#### 5.3.2. *Sea buckthorn* (SBT)

*Hippophae rhamnoides* L., commonly known as SBT, is naturally distributed across Asia and Europe. Various parts of this plant have been used in traditional medicine [131]. In vivo study on the protective effect of SBT leaf powder or leaf extract showed the enhanced regenerative changes in Japanese Quail chick’s livers. Furthermore, it was noticed that ALT levels significantly decreased after the supplementation of SBT and they were comparable to control levels [132].

#### 5.3.3. *Nigella sativa* (Black Seed)

*Nigella sativa* is widely used as medicinal plant (e.g., as a significant medication in Indian Ayurveda and Unani) [133]. Its oil protects the liver from OTA-induced damage. Its coadministration with OTA for four weeks helps to maintain the normal structure of hepatocytes and restores the amount of collagen fibers [134].

#### 5.3.4. *Curcuma longa* Extract (Curcumin)

In vivo study, where rats were treated with olive oil with OTA, showed reduced ability against oxidative stress. Whereas, the addition of curcumin increases the activity of antioxidant enzymes such as SOD, catalase (CAT), and GPx. Moreover, curcumin weakens the effect of the toxin on liver inflammation, steatosis, and necrosis [135]. Curcumin decreases nitrosative stress as it lowers the level of NO and iNOS activity in rats’ livers [136].

#### 5.3.5. Fruit Anthocyanin

Cyanidin-3-*O*-β-glucopyranoside (C3G), an anthocyanin family member which naturally occurs in pigmented oranges, blackberries, strawberries, and cranberries, is known for its antioxidant properties. In the study conducted by Guerra et al. [95] effectiveness of C3G in preventing cytotoxicity induced by OTA in the HepG2 cell line was examined. Authors found that pretreatment with C3G led to a significant reduction in OTA-induced cytotoxicity. Furthermore, C3G was able to significantly decrease ROS generation caused by the toxin at a concentration of 50 µM. The study also showed that C3G had a positive impact on protein and total DNA synthesis, as OTA’s inhibitory effect was reduced. Additionally, C3G exhibited an anti-apoptotic effect by reducing DNA fragmentation and inhibiting caspase-3 activation [95]. This compound was also used in the study performed by Sorrenti et al. [137]. After four weeks of daily treatment, rats with OTA and OTA+C3G showed significant differences. A group treated with both compounds had significantly lower iNOS expression in the liver in comparison to group treated with the toxin only. The presence of C3G decreased the expression of the DDAH-1 protein but did not affect eNOS expression [137].

#### 5.3.6. Yemeni Green Coffee

Yemeni green coffee is a source of caffeoyl-quinic acids one of the intense antioxidative substance. According to report by Nogaim et al. [138], Yemeni green coffee powder can protect the rats’ livers from oxidative stress caused by toxin. OTA reduces the levels of GSH, SOD, CAT, and GR, while coffee increases their activity by increasing their levels. In addition, it was noted that animals treated with OTA in combination with coffee had lower MDA levels in livers, the end product of lipid peroxidation, compared to animals treated with the toxin alone [138].

#### 5.3.7. Green Tea

Green tea-mediated zinc oxide nanoparticles (ZnONPs) appear to be hepatoprotective in albino rats by reducing the lethal effect of OTA. The presence of OTA damages liver cells and leads to necrosis. As a result, the levels of biomarkers associated with liver function, such as ALT and AST, tend to rise. The use of ZnONPs has significantly improved the ALT and AST parameters by decreasing their levels. Furthermore, it was observed the improvement during histopathological examination. The animals that received a combination of OTA and ZnONPs exhibited moderate degeneration in their livers, whereas those who consumed only the toxin experienced severe alterations [139].

#### 5.3.8. Lycopene

Palabiyik et al. reported a significant decrease in selenium, zinc, and copper levels in OTA-treated rats. The addition of lycopene did not affect the liver zinc level, whereas it markedly increased selenium and copper compared to the group treated with OTA only [140]. While OTA increased the number of apoptotic hepatocytes in mice to 97.20 ± 17.09, lycopene could reduce their number to 8.80 ± 5.02 [121]. The commet assay results show the genotoxic effect of OTA as it damaged DNA in the rat livers by increasing the length, moment and tail intensity. The administration of lycopene for 7 and 14 days significantly improved these parameters by decreasing them in comparison to the OTA group. Longer administration of lycopene led to better liver DNA protection as it lowered the parameters by 40%, 66%, and 51% in tail length, tail moment and tail intensity, respectively [141].

#### 5.3.9. Silymarin (Sil)

*Silybum marianum* (milk thistle) is a plant that is native to the Mediterranean region and is now grown and cultivated worldwide [142]. Sil is a flavonoligan extracted from this plant significantly decreased levels of ALT and AST. It was observed after additional Sil supplementation (OTA+Sil) in comparison to supplementation with OTA only chicks [143] and primary chicken hepatocytes [144]. OTA caused granular or vacuolar degeneration in hepatocytes, activation of Kupffer’s cells or capillary endothelium, and hyperemia or perivascular mononuclear infiltration. Sil could alleviate or eliminate these changes in the livers [143]. The in vitro study showed that Sil decreased the apoptosis rate since it decreased Caspase-3 mRNA and increased both Bcl-2 and Bax mRNA expressions. The compound had also a positive impact on the antioxidant effect as it significantly increased SOD activity and GSH levels and decreased MDA levels [144].

#### 5.3.10. Silibinin

Silibinin possess a protective effect on rats’ isolated perfused livers and Kupffer cells against OTA toxicity. It had the inhibitory effect on OTA-mediated TNF-α release. Moreover, silibinin completely restored glutamate dehydrogenase (GLDH) and lactate dehydrogenase (LDH) levels [116]. According to study from 2012, silibinin has been found to be an effective protectant against apoptosis in cultured primary rat hepatocytes. Concentration at 130 µmol/L completely prevented the activation of caspase-3 caused by OTA [145].

### 5.4. Organic Substances

The results obtained after coadministration with OTA three different compounds: ammonium glycyrrhizin (CAG), L-arginine (L-Arg), and glucurolactone (GA) were similar to results with Sil. The apoptotic rates were decreased after their pretreatment to 14.90 ± 1.50, 18.76 ± 1.55 and 27.50 ± 2.16%, respectively. SOD activity and GSH levels were significantly higher comparing to group with OTA only. Moreover, the MDA levels and Caspase-3 mRNA expression were markedly decreased while Bcl-2 mRNA expression was significantly increased after the pretreatment with CAG, L-Arg and GA [144].These compounds certainly possess a positive effect on the liver, protecting it from the OTA toxic effect, which is confirmed by numerous studies. Their action is based on the antioxidant function, thus reducing oxidative stress in cells and lipid peroxidation, and increasing antioxidant enzymes activity. Moreover, they regulate the activity of liver enzymes, such as ALT, AST and ALP. Therefore, it suggests a protective effect of these compounds against one of the main toxic effects of OTA on the liver.

The results indicate the similarities in the action of individual compounds. However, what should be taken into account, all of these compounds are able to mitigate the effects of ochratoxin A, although they are not drugs that will completely eliminate its toxic effect.

## 6. Conclusions

Many studies indicate that OTA contamination in food products is still a prevalent and inevitable problem. This poses a serious threat. Exposing people to the toxin ingestion will result in the occurrence of health problems related to the hepatotoxic effect of OTA. Based on the available experimental studies, it can be suggested that the basic mechanism responsible for hepatocytes damage (including activation of apoptosis, disturbances in biochemical pathways and mitochondria, as well as DNA damage) is related to the induction of oxidative stress. The disturbed balance between free radicals and antioxidants leads to significant hepatocytes damage and significantly affects the metabolism of these cells. Unfortunately, there are many unresolved issues and unanswered questions in the field of the effects of OTA on hepatocytes. The following questions were created due to the discrepancy among results in the above-mentioned studies: Does OTA/OTA-HSA affect PEPCK activity in liver? Does PFKL and PFGK1 expression changes affect glycolysis in liver? What are the molecular differences among described pathways between kidney and liver? What is the reason of different functioning processes after OTA administration between liver and kidneys? To answer these questions, the detailed analysis of gluconeogenesis, glycolysis, PPAR signaling pathway and overall multi-omics should be performed on different cell types.

The results of the conducted experiments clearly indicate that OTA has a detrimental effect on hepatocytes. However, existing analyzes are often performed on different cell lines or organisms, with different incubation times, concentrations, and routes of OTA administration. These discrepancies significantly affect the proposed assumptions, as not all findings can be extrapolated to the human model. One of the main issues is a lack of studies on the human organism, which obstruct the comparison of obtained results concerning whether the impact of OTA in an animal model will have the same molecular background in the case of humans. An example is the ability of OTA to perfuse organs by binding to HSA. In rats, plasma albumin was shown to have a structural and quantitative differences in the binding sites and in various stereoselective behavior than in HSA [146]. Moreover, in animal model studies, the OTA binding process with serum albumin was not evaluated. The OTA influence on hepatotoxicity was performed on various animals, including ducks, rats, and mice. For more reliable observations and conclusions, a specific model (a type of animal, species, doses etc.) that could be replicated in OTA-focused research (as well as enabling a possibility to create a detailed meta-analysis) should be created. Furthermore, different animal models as well as cell lines may express different genes and differ in physiology (e.g., lack of genes expression and proteins associated with metabolic phases). Moreover, bioavailability among animal models also may significantly differ.

A very promising tool for future studies on human liver is the liver-on-a-chip (LOC) device. This technology provides the application of various cell types co-culturing that build human organs. Furthermore, used microfluidic device enable control of fluid flow. Thus, the microenvironment of the cultured cells can be strictly monitored and easily changed [147]. Results obtained from LOC could give very interesting and promising results, especially in the field of liver and drug metabolism.

In addition, the results also show that there are compounds that can positively influence by minimization of the toxic effects of OTA and thus protect the hepatocytes. However, most of the available data as it was mentioned are based on animals, so they do not provide clear answers as they would not be equally effective in humans. For this reason, there is still a need for broader research that could explain the molecular mechanisms of OTA-induced hepatotoxicity, and further research on hepatocytes protective compounds. The published results indicate the relationship between the protective effect on the liver against the toxic effects of OTA and antioxidants, which should be taken into account when taking further research steps.

## Data Availability

Not applicable.

## Abbreviations

ABCC: ATP Binding Cassette Subfamily C; ALP: alkaline phosphatase; ALT: alanine aminotransferase; AST: aspartate aminotransferase; CAG: ammonium glycyrrhizin; CAT: catalase; CD36: Cluster of Differentiation 36; CK: creatinine kinase; CLF: clofibrate; CoQ_10_: Coenzyme Q_10_; COX-2: cyclooxygenase-2; CYP: cytochrome; CYP450: cytochrome P450; C3G: cyanidin-3-*O*-β-d-glucpyranoside; DXM: dexamethasone; FABP2: Fatty Acid Binding Protein 2; GA: glucurolactone; GCL: glutamate-cysteine ligase; G-GT: G-glutamyl transferase; GLDH: glutamate dehydrogenase; GPx: glutathione peroxidase; GR: glutathione reductase; GSH: glutathione; GST: glutathione-S-transferase; HO-1: heme oxygenase-1; HSA: human serum albumin; Hyp: hydroxyproline; ICAD-L: long isoform of inhibitor of caspase-activated DNase; INH: Isoniazid; ISF: isosafrole; L-Arg: L-arginine; LPS: lipopolysaccharide; MEL: melatonin; MDA: malondialdehyde; MT1A: metallothionein1-A; MT2A: metallothionein2-A; NO: nitric oxide; OATs: organic anion transporters; OATPs: organic anion-transporting polypeptides; OTA: ochratoxin A; OTA-: monoanionic ochratoxin A; OTA2-: dianionic ochratoxin A; OTB: ochratoxin B; OTC: Ochratoxin C; OTAα: Ochratoxin α; OTQ: quinone; OTHQ: hydroquinone; PARP: Poly(ADP-ribose) Polymerase; PB: phenobarbital; PCP: pentachlorophenol; PEPCK: phosphoenolpyruvate carboxykinase; PKS: polyketide synthase; PPAR: peroxisome proliferator-activated receptor; PPARα: peroxisome proliferator-activated receptor α; PPARβ/δ: peroxisome proliferator-activated recetor β/δ; PPARγ: peroxisome proliferator-activated receptor γ; PTPC: permeability transition pore complex; ROS: reactive oxygen species; SBT: Sea buckthorn; SeMet: selenomethionine; Sil: silymarin; SOD: superoxide dismutase; SOD1: superoxide dismutase 1; TCBQ: tetrachlorobenzoquinone; ZnONPs: zinc oxide nanoparticles; 3MC: 3-methylcolcanthrene, 4-OH OTA: 4-hydroxyochratoxin A.

## References

[1] Ringot D., Chango A., Schneider Y.J., Larondelle Y. (2006). Toxicokinetics and toxicodynamics of ochratoxin A, an update. Chem. Biol. Interact..

[2] van der Merwe K.J., Steyn P.S., Fourie L., Scott D.B., Theron J.J. (1965). Ochratoxin A, a toxic metabolite produced by Aspergillus ochraceus Wilh. Nature.

[3] Wawrzyniak J., Waśkiewicz A. (2014). Ochratoxin A and citrinin production by Penicillium verrucosum on cereal solid substrates. Food Addit. Contam. Part A.

[4] Harwig J., Chen Y.K. (1974). Some conditions favoring production of ochratoxin a and citrinin by penicillium viridicatum in wheat and barley. Can. J. Plant Sci..

[5] Palacios-Cabrera H., Taniwaki M.H., Hashimoto J.M., Menezes H.C.D. (2005). Growth of Aspergillus ochraceus, A. carbonarius and A. niger on culture media at different water activities and temperatures. Braz. J. Microbiol..

[6] Oliveira G., Evangelista S.R., Passamani F.R.F., Santiago W.D., Cardoso M.D.G., Batista L.R. (2019). Influence of temperature and water activity on Ochratoxin A production by Aspergillus strain in coffee south of Minas Gerais/Brazil. LWT.

[7] Alborch L., Bragulat M.R., Abarca M.L., Cabañes F.J. (2011). Effect of water activity, temperature and incubation time on growth and ochratoxin A production by Aspergillus niger and Aspergillus carbonarius on maize kernels. Int. J. Food Microbiol..

[8] Zebiri S., Mokrane S., Verheecke-Vaessen C., Choque E., Reghioui H., Sabaou N., Mathieu F., Riba A. (2019). Occurrence of ochratoxin A in Algerian wheat and its milling derivatives. Toxin Rev..

[9] Torović L. (2018). Aflatoxins and ochratoxin A in flour: A survey of the Serbian retail market. Food Addit. Contam. Part B Surveill..

[10] Kosicki R., Twarużek M., Dopierała P., Rudzki B., Grajewski J. (2020). Occurrence of Mycotoxins in Winter Rye Varieties Cultivated in Poland (2017–2019). Toxins.

[11] Wajih Ul Hassan S., Sadef Y., Hussain S., Rafique Asi M., Ashraf M.Y., Anwar S., Malik A. (2020). Unusual pattern of aflatoxins and ochratoxin in commercially grown maize varieties of Pakistan. Toxicon.

[12] Iqbal S.Z., Asi M.R., Hanif U., Zuber M., Jinap S. (2016). The presence of aflatoxins and ochratoxin A in rice and rice products; and evaluation of dietary intake. Food Chem..

[13] Lai X., Liu R., Ruan C., Zhang H., Liu C. (2015). Occurrence of aflatoxins and ochratoxin A in rice samples from six provinces in China. Food Control.

[14] Leoni L.A., Soares L.M., Oliveira P.L. (2000). Ochratoxin A in Brazilian roasted and instant coffees. Food Addit. Contam..

[15] Galarce-Bustos O., Alvarado M., Vega M., Aranda M. (2014). Occurrence of ochratoxin A in roasted and instant coffees in Chilean market. Food Control.

[16] Juan C., Raiola A., Mañes J., Ritieni A. (2014). Presence of mycotoxin in commercial infant formulas and baby foods from Italian market. Food Control.

[17] Darouj E., Massouh L., Ghanem I. (2016). Investigation of ochratoxin A in Syrian consumed baby foods. Food Control.

[18] Al-Taher F., Cappozzo J., Zweigenbaum J., Lee H.J., Jackson L., Ryu D. (2017). Detection and quantitation of mycotoxins in infant cereals in the U.S. market by LC-MS/MS using a stable isotope dilution assay. Food Control.

[19] Iqbal S.Z., Mehmood Z., Asi M.R., Shahid M., Sehar M., Malik N. (2018). Co-occurrence of aflatoxins and ochratoxin A in nuts, dry fruits, and nuty products. J. Food Saf..

[20] Silva A., Fungaro M.H.P., Silva J.J., Martins L.M., Taniwaki M.H., Iamanaka B.T. (2021). Ochratoxin A and related fungi in Brazilian black pepper (*Piper nigrum* L.). Food Res. Int..

[21] El Darra N., Gambacorta L., Solfrizzo M. (2019). Multimycotoxins occurrence in spices and herbs commercialized in Lebanon. Food Control.

[22] Nguegwouo E., Sone L.E., Tchuenchieu A., Tene H.M., Mounchigam E., Njayou N.F., Nama G.M. (2018). Ochratoxin A in black pepper, white pepper and clove sold in Yaoundé (Cameroon) markets: Contamination levels and consumers’ practices increasing health risk. Int. J. Food Contam..

[23] Iqbal S.Z., Asi M.R., Zuber M., Akhtar J., Jawwad Saif M. (2013). Natural occurrence of aflatoxins and ochratoxin A in commercial chilli and chilli sauce samples. Food Control.

[24] Iqbal S.Z., Mumtaz A., Mahmood Z., Waqas M., Ghaffar A., Ismail A., Pervaiz W. (2021). Assessment of aflatoxins and ochratoxin a in chili sauce samples and estimation of dietary intake. Food Control.

[25] Remiro R., Irigoyen A., González-Peñas E., Lizarraga E., López de Cerain A. (2013). Levels of ochratoxins in Mediterranean red wines. Food Control.

[26] Silva L.J.G., Teixeira A.C., Pereira A.M.P.T., Pena A., Lino C.M. (2020). Ochratoxin A in Beers Marketed in Portugal: Occurrence and Human Risk Assessment. Toxins.

[27] Zhang Z., Fan Z., Nie D., Zhao Z., Han Z. (2019). Analysis of the Carry-Over of Ochratoxin A from Feed to Milk, Blood, Urine, and Different Tissues of Dairy Cows Based on the Establishment of a Reliable LC-MS/MS Method. Molecules.

[28] Armorini S., Altafini A., Zaghini A., Roncada P. (2016). Ochratoxin A in artisan salami produced in Veneto (Italy). Food Addit. Contam. Part B Surveill..

[29] Altafini A., Fedrizzi G., Roncada P. (2019). Occurrence of ochratoxin A in typical salami produced in different regions of Italy. Mycotoxin Res..

[30] Roncada P., Altafini A., Fedrizzi G., Guerrini A., Polonini G.L., Elisabetta C. (2020). Ochratoxin A contamination of the casing and the edible portion of artisan salamis produced in two Italian regions. World Mycotoxin J..

[31] Iqbal S.Z., Nisar S., Asi M.R., Jinap S. (2014). Natural incidence of aflatoxins, ochratoxin A and zearalenone in chicken meat and eggs. Food Control.

[32] Turkoglu C., Keyvan E. (2019). Determination of Aflatoxin M1 and Ochratoxin A in Raw, Pasteurized and UHT Milk in Turkey. Acta Sci. Vet..

[33] Altafini A., Roncada P., Guerrini A., Minkoumba Sonfack G., Fedrizzi G., Caprai E. (2021). Occurrence of Ochratoxin A in Different Types of Cheese Offered for Sale in Italy. Toxins.

[34] Cramer B., Osteresch B., Muñoz K.A., Hillmann H., Sibrowski W., Humpf H.U. (2015). Biomonitoring using dried blood spots: Detection of ochratoxin A and its degradation product 2’R-ochratoxin A in blood from coffee drinkers. Mol. Nutr. Food Res..

[35] Biasucci G., Calabrese G., Di Giuseppe R., Carrara G., Colombo F., Mandelli B., Maj M., Bertuzzi T., Pietri A., Rossi F. (2011). The presence of ochratoxin A in cord serum and in human milk and its correspondence with maternal dietary habits. Eur. J. Nutr..

[36] Solfrizzo M., Gambacorta L., Visconti A. (2014). Assessment of Multi-Mycotoxin Exposure in Southern Italy by Urinary Multi-Biomarker Determination. Toxins.

[37] Ali N., Muñoz K., Degen G.H. (2017). Ochratoxin A and its metabolites in urines of German adults-An assessment of variables in biomarker analysis. Toxicol. Lett..

[38] Muñoz K., Cramer B., Dopstadt J., Humpf H.U., Degen G.H. (2017). Evidence of ochratoxin A conjugates in urine samples from infants and adults. Mycotoxin Res..

[39] Klarić M.Š., Rašić D., Peraica M. (2013). Deleterious Effects of Mycotoxin Combinations Involving Ochratoxin A. Toxins.

[40] Ostry V., Malir F., Toman J., Grosse Y. (2017). Mycotoxins as human carcinogens-the IARC Monographs classification. Mycotoxin Res..

[41] EC (2023). Commission Regulation (EU) 2023/915 of 25 April 2023 on maximum levels for certain contaminants in food and repealing Regulation (EC) No 1881/2006. Off. J. Eur. Union.

[42] Kőszegi T., Poór M. (2016). Ochratoxin A: Molecular Interactions, Mechanisms of Toxicity and Prevention at the Molecular Level. Toxins.

[43] El Khoury A., Atoui A. (2010). Ochratoxin A: General Overview and Actual Molecular Status. Toxins.

[44] Ihlenfeldt W.D., Bolton E.E., Bryant S.H. (2009). The PubChem chemical structure sketcher. J. Cheminform..

[45] Maor U., Barda O., Sadhasivam S., Bi Y., Levin E., Zakin V., Prusky D.B., Sionov E. (2021). Functional roles of LaeA, polyketide synthase, and glucose oxidase in the regulation of ochratoxin A biosynthesis and virulence in Aspergillus carbonarius. Mol. Plant Pathol..

[46] Zhang J., Zhu L., Chen H., Li M., Zhu X., Gao Q., Wang D., Zhang Y. (2016). A Polyketide Synthase Encoded by the Gene An15g07920 Is Involved in the Biosynthesis of Ochratoxin A in Aspergillus niger. J. Agric. Food Chem..

[47] Bacha N., Atoui A., Mathieu F., Liboz T., Lebrihi A. (2009). Aspergillus westerdijkiae polyketide synthase gene “aoks1” is involved in the biosynthesis of ochratoxin A. Fungal Genet. Biol..

[48] Färber P., Geisen R. (2004). Analysis of Differentially-Expressed Ochratoxin A Biosynthesis Genes of Penicillium Nordicum. Eur. J. Plant Pathol..

[49] Rodríguez A., Medina Á., Córdoba J.J., Magan N. (2014). The influence of salt (NaCl) on ochratoxin A biosynthetic genes, growth and ochratoxin A production by three strains of Penicillium nordicum on a dry-cured ham-based medium. Int. J. Food Microbiol..

[50] Ferrara M., Gallo A., Cervini C., Gambacorta L., Solfrizzo M., Baker S.E., Perrone G. (2021). Evidence of the Involvement of a Cyclase Gene in the Biosynthesis of Ochratoxin A in Aspergillus carbonarius. Toxins.

[51] Wei S., Hu C., Zhang Y., Lv Y., Zhang S., Zhai H., Hu Y. (2023). AnAzf1 acts as a positive regulator of ochratoxin A biosynthesis in Aspergillus niger. Appl. Microbiol. Biotechnol..

[52] Gerin D., Garrapa F., Ballester A.-R., González-Candelas L., De Miccolis Angelini R.M., Faretra F., Pollastro S. (2021). Functional Role of Aspergillus carbonarius AcOTAbZIP Gene, a bZIP Transcription Factor within the OTA Gene Cluster. Toxins.

[53] Gallo A., Bruno K.S., Solfrizzo M., Perrone G., Mulè G., Visconti A., Baker S.E. (2012). New insight into the ochratoxin A biosynthetic pathway through deletion of a nonribosomal peptide synthetase gene in Aspergillus carbonarius. Appl. Environ. Microbiol..

[54] Crespo-Sempere A., Marín S., Sanchis V., Ramos A.J. (2013). VeA and LaeA transcriptional factors regulate ochratoxin A biosynthesis in Aspergillus carbonarius. Int. J. Food Microbiol..

[55] Zhang J., Chen H., Sumarah M.W., Gao Q., Wang D., Zhang Y. (2018). veA Gene Acts as a Positive Regulator of Conidia Production, Ochratoxin A Biosynthesis, and Oxidative Stress Tolerance in Aspergillus niger. J. Agric. Food Chem..

[56] Hagelberg S., Hult K., Fuchs R. (1989). Toxicokinetics of ochratoxin A in several species and its plasma-binding properties. J. Appl. Toxicol..

[57] Studer-Rohr I., Schlatter J., Dietrich D.R. (2000). Kinetic parameters and intraindividual fluctuations of ochratoxin A plasma levels in humans. Arch. Toxicol..

[58] Kane A., Creppy E.E., Roth A., Röschenthaler R., Dirheimer G. (1986). Distribution of the [3H]-label from low doses of radioactive ochratoxin A ingested by rats, and evidence for DNA single-strand breaks caused in liver and kidneys. Arch. Toxicol..

[59] Zimmerli B., Dick R. (1995). Determination of ochratoxin A at the ppt level in human blood, serum, milk and some foodstuffs by high-performance liquid chromatography with enhanced fluorescence detection and immunoaffinity column cleanup: Methodology and Swiss data. J. Chromatogr. B Biomed. Sci. Appl..

[60] Jung K.Y., Takeda M., Kim D.K., Tojo A., Narikawa S., Yoo B.S., Hosoyamada M., Cha S.H., Sekine T., Endou H. (2001). Characterization of ochratoxin A transport by human organic anion transporters. Life Sci..

[61] Bahnemann E., Kerling H.P., Ensminger S., Schwerdt G., Silbernagl S., Gekle M. (1997). Renal transepithelial secretion of ochratoxin A in the non-filtering toad kidney. Toxicology.

[62] Leier I., Hummel-Eisenbeiss J., Cui Y., Keppler D. (2000). ATP-dependent para-aminohippurate transport by apical multidrug resistance protein MRP2. Kidney Int..

[63] Kontaxi M., Echkardt U., Hagenbuch B., Stieger B., Meier P.J., Petzinger E. (1996). Uptake of the mycotoxin ochratoxin A in liver cells occurs via the cloned organic anion transporting polypeptide. J. Pharmacol. Exp. Ther..

[64] Wang J., Gan C., Qi X., Lebre M.C., Schinkel A.H. (2020). Human organic anion transporting polypeptide (OATP) 1B3 and mouse OATP1A/1B affect liver accumulation of Ochratoxin A in mice. Toxicol. Appl. Pharmacol..

[65] Størmer F.C., Hansen C.E., Pedersen J.I., Hvistendahl G., Aasen A.J. (1981). Formation of (4R)- and (4S)-4-hydroxyochratoxin A from ochratoxin A by liver microsomes from various species. Appl. Environ. Microbiol..

[66] Størmer F.C., Støren O., Hansen C.E., Pedersen J.I., Aasen A.J. (1983). Formation of (4R)- and (4S)-4-hydroxyochratoxin A and 10-hydroxyochratoxin A from Ochratoxin A by rabbit liver microsomes. Appl. Environ. Microbiol..

[67] Omar R.F., Gelboin H.V., Rahimtula A.D. (1996). Effect of cytochrome P450 induction on the metabolism and toxicity of ochratoxin A. Biochem. Pharmacol..

[68] Ueno Y. (1985). Biotransformation of Mycotoxins in the Reconstituted Cytochrome P-450 System. Mycotoxins.

[69] Gross-Steinmeyer K., Weymann J., Hege H.G., Metzler M. (2002). Metabolism and lack of DNA reactivity of the mycotoxin ochratoxin a in cultured rat and human primary hepatocytes. J. Agric. Food Chem..

[70] Yang S., Zhang H., De Saeger S., De Boevre M., Sun F., Zhang S., Cao X., Wang Z. (2015). In vitro and in vivo metabolism of ochratoxin A: A comparative study using ultra-performance liquid chromatography-quadrupole/time-of-flight hybrid mass spectrometry. Anal. Bioanal. Chem..

[71] Gupta R.C., Gupta R.C. (2012). Chapter 91—Ochratoxins and citrinin. Veterinary Toxicology.

[72] Pfohl-Leszkowicz A., Manderville R.A. (2007). Ochratoxin A: An overview on toxicity and carcinogenicity in animals and humans. Mol. Nutr. Food Res..

[73] Gayathri L., Dhivya R., Dhanasekaran D., Periasamy V.S., Alshatwi A.A., Akbarsha M.A. (2015). Hepatotoxic effect of ochratoxin A and citrinin, alone and in combination, and protective effect of vitamin E: In vitro study in HepG2 cell. Food Chem. Toxicol..

[74] Shin H.S., Lee H.J., Pyo M.C., Ryu D., Lee K.-W. (2019). Ochratoxin A-Induced Hepatotoxicity through Phase I and Phase II Reactions Regulated by AhR in Liver Cells. Toxins.

[75] Qi X., Yang X., Chen S., He X., Dweep H., Guo M., Cheng W.-H., Xu W., Luo Y., Gretz N. (2014). Ochratoxin A induced early hepatotoxicity: New mechanistic insights from microRNA, mRNA and proteomic profiling studies. Sci. Rep..

[76] Consortium T.U. (2018). UniProt: A worldwide hub of protein knowledge. Nucleic Acids Res..

[77] Wang W., Zhai S., Xia Y., Wang H., Ruan D., Zhou T., Zhu Y., Zhang H., Zhang M., Ye H. (2019). Ochratoxin A induces liver inflammation: Involvement of intestinal microbiota. Microbiome.

[78] Nuttall F.Q., Ngo A., Gannon M.C. (2008). Regulation of hepatic glucose production and the role of gluconeogenesis in humans: Is the rate of gluconeogenesis constant?. Diabetes Metab. Res. Rev..

[79] She P., Shiota M., Shelton K.D., Chalkley R., Postic C., Magnuson M.A. (2000). Phosphoenolpyruvate carboxykinase is necessary for the integration of hepatic energy metabolism. Mol. Cell. Biol..

[80] Meisner H., Meisner P. (1981). Ochratoxin A, an in vivo inhibitor of renal phosphoenolpyruvate carboxykinase. Arch. Biochem. Biophys..

[81] Il’ichev Y.V., Perry J.L., Simon J.D. (2002). Interaction of Ochratoxin A with Human Serum Albumin. Preferential Binding of the Dianion and pH Effects. J. Phys. Chem. B.

[82] Il’ichev Y.V., Perry J.L., Rüker F., Dockal M., Simon J.D. (2002). Interaction of ochratoxin A with human serum albumin. Binding sites localized by competitive interactions with the native protein and its recombinant fragments. Chem. Biol. Interact..

[83] Hundhausen C., Boesch-Saadatmandi C., Matzner N., Lang F., Blank R., Wolffram S., Blaschek W., Rimbach G. (2008). Ochratoxin a lowers mRNA levels of genes encoding for key proteins of liver cell metabolism. Cancer Genom. Proteom..

[84] Ahmadian M., Suh J.M., Hah N., Liddle C., Atkins A.R., Downes M., Evans R.M. (2013). PPARγ signaling and metabolism: The good, the bad and the future. Nat. Med..

[85] Wang Y., Nakajima T., Gonzalez F.J., Tanaka N. (2020). PPARs as Metabolic Regulators in the Liver: Lessons from Liver-Specific PPAR-Null Mice. Int. J. Mol. Sci..

[86] Berthier A., Johanns M., Zummo F.P., Lefebvre P., Staels B. (2021). PPARs in liver physiology. Biochim. Biophys. Acta Mol. Basis Dis..

[87] Lee Y.K., Park J.E., Lee M., Hardwick J.P. (2018). Hepatic lipid homeostasis by peroxisome proliferator-activated receptor gamma 2. Liver Res..

[88] Zheng Q.W., Ding X.F., Cao H.J., Ni Q.Z., Zhu B., Ma N., Zhang F.K., Wang Y.K., Xu S., Chen T.W. (2021). Ochratoxin A Induces Steatosis via PPARγ-CD36 Axis. Toxins.

[89] Szklarczyk D., Gable A.L., Nastou K.C., Lyon D., Kirsch R., Pyysalo S., Doncheva N.T., Legeay M., Fang T., Bork P. (2021). The STRING database in 2021: Customizable protein-protein networks, and functional characterization of user-uploaded gene/measurement sets. Nucleic Acids Res..

[90] Choi S.Y., Kim T.H., Hong M.W., Park T.S., Lee H., Lee S.J. (2020). Transcriptomic alterations induced by aflatoxin B1 and ochratoxin A in LMH cell line. Poult. Sci..

[91] Omar R.F., Hasinoff B.B., Mejilla F., Rahimtula A.D. (1990). Mechanism of ochratoxin a stimulated lipid peroxidation. Biochem. Pharmacol..

[92] Manderville R., Leszkowicz A. (2006). Chapter 4 Genotoxicity of Chlorophenols and Ochratoxin A. Adv. Mol. Toxicol..

[93] Hoehler D., Marquardt R.R., McIntosh A.R., Hatch G.M. (1997). Induction of free radicals in hepatocytes, mitochondria and microsomes of rats by ochratoxin A and its analogs. Biochim. Biophys. Acta Mol. Cell. Res..

[94] Atroshi F., Biese I., Saloniemi H., Ali-Vehmas T., Saari S., Rizzo A., Veijalainen P. (2000). Significance of apoptosis and its relationship to antioxidants after ochratoxin A administration in mice. J. Pharm. Pharm. Sci..

[95] Guerra M.C., Galvano F., Bonsi L., Speroni E., Costa S., Renzulli C., Cervellati R. (2005). Cyanidin-3-O-β-glucopyranoside, a natural free-radical scavenger against aflatoxin B1- and ochratoxin A-induced cell damage in a human hepatoma cell line (Hep G2) and a human colonic adenocarcinoma cell line (CaCo-2). Br. J. Nutr..

[96] Domijan A.M., Rudes K., Peraica M. (2005). The effect of ochratoxin A on the concentration of protein carbonyls in rats. Arch. Ind. Hyg. Toxicol..

[97] Marin-Kuan M., Nestler S., Verguet C., Bezençon C., Piguet D., Mansourian R., Holzwarth J., Grigorov M., Delatour T., Mantle P. (2006). A toxicogenomics approach to identify new plausible epigenetic mechanisms of ochratoxin a carcinogenicity in rat. Toxicol. Sci..

[98] Cavin C., Delatour T., Marin-Kuan M., Holzhäuser D., Higgins L., Bezençon C., Guignard G., Junod S., Richoz-Payot J., Gremaud E. (2007). Reduction in antioxidant defenses may contribute to ochratoxin A toxicity and carcinogenicity. Toxicol. Sci..

[99] Zhang Z., Xu J., Zhang X., Wang J., Xie H., Sun Y., Zhang Q., Chang Z., Liu Y. (2022). Protective Effect of SeMet on Liver Injury Induced by Ochratoxin A in Rabbits. Toxins.

[100] Kanehisa M., Goto S. (2000). KEGG: Kyoto encyclopedia of genes and genomes. Nucleic Acids Res..

[101] Huang D.W., Sherman B.T., Lempicki R.A. (2009). Bioinformatics enrichment tools: Paths toward the comprehensive functional analysis of large gene lists. Nucleic Acids Res..

[102] Sherman B.T., Hao M., Qiu J., Jiao X., Baseler M.W., Lane H.C., Imamichi T., Chang W. (2022). DAVID: A web server for functional enrichment analysis and functional annotation of gene lists (2021 update). Nucleic Acids Res..

[103] Parvataneni S., Vemuri-Reddy S. (2020). N-acetyl Cysteine Use in the Treatment of Shock Liver. Cureus.

[104] Sueck F., Specht J., Cramer B., Humpf H.U. (2020). Identification of ochratoxin-N-acetyl-L-cysteine as a new ochratoxin A metabolite and potential biomarker in human urine. Mycotoxin Res..

[105] Yang Q., Shi L., Huang K., Xu W. (2014). Protective effect of N-acetylcysteine against DNA damage and S-phase arrest induced by ochratoxin A in human embryonic kidney cells (HEK-293). Food Chem. Toxicol..

[106] Pfohl-Leszkowicz A., Manderville R.A. (2012). An update on direct genotoxicity as a molecular mechanism of ochratoxin a carcinogenicity. Chem. Res. Toxicol..

[107] Ma T.-F., Chen Y.-P., Shen Y., Chen Y.-P., Ma T.-F. (2021). Chapter Seven—Progress in the applications of surface plasmon resonance for food safety. Comprehensive Analytical Chemistry.

[108] Waidyanatha S., Lin P.H., Rappaport S.M. (1996). Characterization of chlorinated adducts of hemoglobin and albumin following administration of pentachlorophenol to rats. Chem. Res. Toxicol..

[109] Vaidyanathan V.G., Villalta P.W., Sturla S.J. (2007). Nucleobase-dependent reactivity of a quinone metabolite of pentachlorophenol. Chem. Res. Toxicol..

[110] Murray A.R., Kisin E., Castranova V., Kommineni C., Gunther M.R., Shvedova A.A. (2007). Phenol-induced in vivo oxidative stress in skin: Evidence for enhanced free radical generation, thiol oxidation, and antioxidant depletion. Chem. Res. Toxicol..

[111] Dai J., Park G., Wright M.W., Adams M., Akman S.A., Manderville R.A. (2002). Detection and characterization of a glutathione conjugate of ochratoxin A. Chem. Res. Toxicol..

[112] Gillman I.G., Clark T.N., Manderville R.A. (1999). Oxidation of ochratoxin A by an Fe-porphyrin system: Model for enzymatic activation and DNA cleavage. Chem. Res. Toxicol..

[113] Kamp H.G., Eisenbrand G., Janzowski C., Kiossev J., Latendresse J.R., Schlatter J., Turesky R.J. (2005). Ochratoxin A induces oxidative DNA damage in liver and kidney after oral dosing to rats. Mol. Nutr. Food Res..

[114] Chopra M., Link P., Michels C., Schrenk D. (2010). Characterization of ochratoxin A-induced apoptosis in primary rat hepatocytes. Cell Biol. Toxicol..

[115] Bouaziz C., Sharaf El Dein O., El Golli E., Abid-Essefi S., Brenner C., Lemaire C., Bacha H. (2008). Different apoptotic pathways induced by zearalenone, T-2 toxin and ochratoxin A in human hepatoma cells. Toxicology.

[116] Al-Anati L., Essid E., Reinehr R., Petzinger E. (2009). Silibinin protects OTA-mediated TNF-alpha release from perfused rat livers and isolated rat Kupffer cells. Mol. Nutr. Food Res..

[117] Barré-Sinoussi F., Montagutelli X. (2015). Animal models are essential to biological research: Issues and perspectives. Future Sci. OA.

[118] Pound P., Ebrahim S., Sandercock P., Bracken M.B., Roberts I. (2004). Where is the evidence that animal research benefits humans?. BMJ.

[119] Hoehler D., Marquardt R.R. (1996). Influence of vitamins E and C on the toxic effects of ochratoxin A and T-2 toxin in chicks. Poult. Sci..

[120] Shalaby A.M.E. The opposing effect of ascorbic acid (vitamin C) on ochratoxin toxicity in nile tilapia (Oreochromis niloticus). Proceedings of the 6th International Symposium on Tilapia in Aquaculture.

[121] Badriyah B., Hastuti U.S. The effect of pomelo citrus (Citrus maxima var. Nambangan), vitamin C and lycopene towards the number reduction of mice (Mus musculus) apoptotic hepatocyte caused of ochratoxin A. Proceedings of the AIP Conference Proceedings.

[122] Ramyaa P., Krishnaswamy R., Padma V.V. (2014). Quercetin modulates OTA-induced oxidative stress and redox signalling in HepG2 cells—Up regulation of Nrf2 expression and down regulation of NF-κB and COX-2. Biochim. Biophys. Acta.

[123] Oršolić N., Jazvinšćak Jembrek M., Terzić S. (2017). Honey and quercetin reduce ochratoxin A-induced DNA damage in the liver and the kidney through the modulation of intestinal microflora. Food Agric. Immunol..

[124] Sutken E., Aral E., Ozdemir F., Uslu S., Alatas O., Colak O. (2007). Protective role of melatonin and coenzyme Q10 in ochratoxin A toxicity in rat liver and kidney. Int. J. Toxicol..

[125] Soyöz M., Ozçelik N., Kilinç I., Altuntaş I. (2004). The effects of ochratoxin A on lipid peroxidation and antioxidant enzymes: A protective role of melatonin. Cell Biol. Toxicol..

[126] Meki A.R., Hussein A.A. (2001). Melatonin reduces oxidative stress induced by ochratoxin A in rat liver and kidney. Comp. Biochem. Physiol. Part C Toxicol. Pharmacol..

[127] Abdel-Wahhab M.A., Abdel-Galil M.M., El-Lithey M. (2005). Melatonin counteracts oxidative stress in rats fed an ochratoxin A contaminated diet. J. Pineal Res..

[128] Xia D., Yang L., Li Y., Chen J., Zhang X., Wang H., Zhai S., Jiang X., Meca G., Wang S. (2021). Melatonin alleviates Ochratoxin A-induced liver inflammation involved intestinal microbiota homeostasis and microbiota-independent manner. J. Hazard. Mater..

[129] Zheng J., Zhang Y., Xu W., Luo Y., Hao J., Shen X.L., Yang X., Li X., Huang K. (2013). Zinc protects HepG2 cells against the oxidative damage and DNA damage induced by ochratoxin A. Toxicol. Appl. Pharmacol..

[130] Chakraborty D., Verma R. (2010). Ameliorative effect of Emblica officinalis aqueous extract on ochratoxin-induced lipid peroxidation in the kidney and liver of mice. Int. J. Occup. Med. Environ. Health.

[131] Guliyev V.B., Gul M., Yildirim A. (2004). *Hippophae rhamnoides* L.: Chromatographic methods to determine chemical composition, use in traditional medicine and pharmacological effects. J. Chromatogr. B Analyt. Technol. Biomed. Life Sci..

[132] Patial V., Asrani R., Patil R., Kumar N., Sharma R. (2015). Protective Effect of Sea buckthorn (*Hippophae rhamnoides* L.) Leaves on Ochratoxin-A Induced Hepatic Injury in Japanese Quail. Vet. Res. Int..

[133] Ahmad A., Husain A., Mujeeb M., Khan S.A., Najmi A.K., Siddique N.A., Damanhouri Z.A., Anwar F. (2013). A review on therapeutic potential of Nigella sativa: A miracle herb. Asian Pac. J. Trop. Biomed..

[134] Alhussaini M., Alyahya A. (2014). Protective Role of Nigella Sativa Oil on Ochratoxin A Toxicity in Liver and Kidney of Male Albino Rats: Histological and Histochemical Studies. Wulfenia.

[135] Damiano S., Longobardi C., Andretta E., Prisco F., Piegari G., Squillacioti C., Montagnaro S., Pagnini F., Badino P., Florio S. (2021). Antioxidative Effects of Curcumin on the Hepatotoxicity Induced by Ochratoxin A in Rats. Antioxidants.

[136] Longobardi C., Damiano S., Andretta E., Prisco F., Russo V., Pagnini F., Florio S., Ciarcia R. (2021). Curcumin Modulates Nitrosative Stress, Inflammation, and DNA Damage and Protects against Ochratoxin A-Induced Hepatotoxicity and Nephrotoxicity in Rats. Antioxidants.

[137] Sorrenti V., Di Giacomo C., Acquaviva R., Bognanno M., Grilli E., D’Orazio N., Galvano F. (2012). Dimethylarginine Dimethylaminohydrolase/Nitric Oxide Synthase Pathway in Liver and Kidney: Protective Effect of Cyanidin 3-O-β-D-Glucoside on Ochratoxin-A Toxicity. Toxins.

[138] Nogaim Q.A., Bugata L.S.P., Prabhakar P.V., Reddy U.A., Kumari I., Mahboob M. (2020). Protective effect of Yemeni green coffee powder against the oxidative stress induced by Ochratoxin A. Toxicol. Rep..

[139] Hassan S., Mujahid H., Ali M.M., Irshad S., Naseer R., Saeed S., Firyal S., Arooj F. (2021). Synthesis, characterization and protective effect of green tea-mediated zinc oxide nanoparticles against ochratoxin A induced hepatotoxicity and nephrotoxicity in albino rats. Appl. Nanosci..

[140] Palabiyik S.S., Erkekoglu P., Kızılgun M., Sahin G., Kocer-Gumusel B. (2017). Lycopene restores trace element levels in ochratoxin A-treated rats. Arch. Ind. Hyg. Toxicol..

[141] Aydin S., Palabiyik S., Erkekoglu P., Sahin G., Başaran N., Giray B.K. (2013). The carotenoid lycopene protects rats against DNA damage induced by Ochratoxin A. Toxicon.

[142] Abenavoli L., Izzo A.A., Milić N., Cicala C., Santini A., Capasso R. (2018). Milk thistle (*Silybum marianum*): A concise overview on its chemistry, pharmacological, and nutraceutical uses in liver diseases. Phytother. Res..

[143] Stoev S., Mircheva T., Denev S., Chobanova S., Ivanov V. (2021). The Protective Effect of Silymarin against Ochratoxin A Induced Histopathological and Biochemical Changes in Chicks. J. Adv. Vet. Res..

[144] Yu Z., Wu F., Tian J., Guo X., An R. (2018). Protective effects of compound ammonium glycyrrhizin, L-arginine, silymarin and glucurolactone against liver damage induced by ochratoxin A in primary chicken hepatocytes. Mol. Med. Rep..

[145] Essid E., Dernawi Y., Petzinger E. (2012). Apoptosis Induction by OTA and TNF-α in Cultured Primary Rat Hepatocytes and Prevention by Silibinin. Toxins.

[146] Massolini G., De Lorenzi E., Ponci M.C., Caccialanza G. (1996). Comparison of drug binding sites on rat and human serum albumins using immobilized-protein stationary phases as a tool for the selection of suitable animal models in pharmacological studies. Boll. Chim. Farm..

[147] Deguchi S., Takayama K. (2022). State-of-the-art liver disease research using liver-on-a-chip. Inflamm. Regen..

